# Meta-analyses of clozapine, norclozapine levels and their ratio across three genome wide association studies

**DOI:** 10.1038/s41398-025-03649-0

**Published:** 2025-10-24

**Authors:** Susanna Maria Rask, Anssi Solismaa, Ari Ahola-Olli, Espen Molden, Kevin Sean O’Connell, Leo-Pekka Lyytikäinen, Nina Mononen, Terho Lehtimäki, Olli Kampman

**Affiliations:** 1 Department of Psychiatry, Faculty of Medicine and Health Technology, Tampere University, Tampere, Finland; 2 Department of Psychiatry, The Pirkanmaa Wellbeing Services County, Tampere, Finland; 3 Cardiac Unit, Department of Internal Medicine, Satasairaala, Pori, Finland; 4 Abomics Ltd, Turku, Finland; 5 Center for Psychopharmacology, Diakonhjemmet Hospital, Oslo, Norway; 6 Centre for Precision Psychiatry, Division of Mental Health and Addiction, Oslo University Hospital & Institute of Clinical Medicine, University of Oslo, Oslo, Norway; 7 Clinical Chemistry, Tampere University Hospital, and Finnish Cardiovascular Research Center-Tampere Faculty of Medicine and Health Technology, Tampere University, Tampere, Finland; 8 Department of Psychiatry, Department of Clinical Sciences (Psychiatry), Faculty of Medicine, University Hospital of Umeå, Umeå University, Umeå, Sweden; 9 Department of Clinical Medicine (Psychiatry), Faculty of Medicine, University of Turku, Turku, Finland; 10 Department of Psychiatry, The Wellbeing Services County of Ostrobothnia, Vaasa, Finland

**Keywords:** Pharmacogenomics, Clinical genetics

## Abstract

Recent genome wide association studies (GWAS) found associations between clozapine serum levels and single nucleotide polymorphisms (SNP) in intragenic region between cytochrome P450 1A1 (*CYP1A1*) and *CYP1A2* and nuclear factor 1B (*NFIB*). The aim of this study was to perform another GWAS of polymorphisms associated with the serum levels of clozapine and norclozapine, their ratios, and to perform meta-analyses with two previous GWAS. Finnish clozapine patients (n = 170) with known smoking habits were genotyped. GWAS was performed with clozapine concentration/dose ratio (C/D), norclozapine C/D and clozapine/norclozapine-ratio as phenotypes, adjusting for age, sex and first four genetic principal components, and additionally for smoking and valproate use. The two other patient populations were from the British CLOZUK2 study (n = 2989) and Norwegian Diakonhjemmet Hospital study (n = 484). In the three population (n = 3643) meta-analyses the top SNP associated with the clozapine C/D ratio was rs2472297, located between the *CYP1A1* and *CYP1A2* genes. For the norclozapine C/D ratio, an association signal was found near uridine-5´-diphospho-glucunorosyltransferase (*UGT*) *UGT2B10* gene. Additionally, rs3732218, an intron variant in the *UGT1A* family gene complex, was associated with norclozapine C/D. Lead SNP associated with the clozapine/norclozapine ratio was rs6827692, an intron variant near *UGT2B7* gene. In the two population meta-analyses (n = 654) adjusting for smoking and valproate use, the *UGT2B10* intron variant rs835309 was associated with the clozapine/norclozapine ratio. We suggest a *UGT2B10* missense SNP rs61750900, in perfect linkage disequilibrium with *UGT2B10* rs835309, as the probable causal variant. Our study confirms and extends the number of genetic variants affecting clozapine and norclozapine metabolism.

## Introduction

Clozapine is the only approved medication for treatment resistant schizophrenia which affects approximately 30% of patients with schizophrenia [1]. Treatment with clozapine is complicated by many factors, the most important being the risk of developing neutropenia or agranulocytosis [2]. Other adverse effects of clozapine treatment, such as hypersalivation [3], weight gain [4], paralytic ileus [5], seizures and risk of diabetes mellitus type II are connected to the serum levels of clozapine and its metabolites [6, 7]. The efficacy of clozapine treatment is tied to serum levels of clozapine and norclozapine [8]. Clozapine metabolism is mainly influenced by patient’s sex, age, ethnicity, weight and smoking status [9]. Main cytochrome P450 enzymes which metabolize clozapine are CYP1A2 and CYP3A4, with the role of CYP1A2 being the most important [10, 11]. In addition, glucuronidation pathways are shown to be of importance for clozapine metabolism and treatment discontinuation, without significantly affecting its serum levels [12, 13]. Interindividual variation in clozapine metabolism has become a point of interest due to the need for safe titration procedures and efficacious but safe management of clozapine serum levels [14].

Earlier genome-wide association study (GWAS) demonstrated that rs2472299-T between *CYP1A1* and *CYP1A2* is associated with decreased serum clozapine concentration [15]. Recently, the role of several SNPs in clozapine metabolism in a cross-ancestral patient sample was investigated and the study identified gene loci in uridine-5´-diphospho-glucunorosyltransferase (*UGT*) UGT1A*, *CYP1A1*/*CYP1A2* and novel SNP in cytochrome P450 oxidoreductase gene (*POR)* that were associated with clozapine metabolism [16]. However, in neither of these studies the association analyses were adjusted with the patients’ smoking status [15, 16].

A GWAS study conducted on Norwegian patients demonstrated that rs28379954-C within nuclear factor 1B (*NFIB)* gene which was associated with 42% reduced dosed adjusted serum concentration of clozapine after accounting for smoking habits [17]. In a subsequent mechanistic study, rs28379954-C accelerated hydroxylation of risperidone in CYP2D6 normal metabolizers up to the level of CYP2D6 ultrarapid metabolizers [18]. This indicates a wider role of NFIB in regulating cytochrome P450 system, thus revealing a perhaps unknown layer of regulation in genetics of drug metabolism. Carriers of both rs28379954-T and rs2472299-T might need considerably larger doses of clozapine to reach therapeutic serum levels [19]. Furthermore, a recent GWAS of clozapine treated patients found a trend of *NFIB* rs1923778 being associated with treatment response without major differences in dose-adjusted clozapine serum levels between genotypes [20].

The aim of this study was to increase understanding from clozapine metabolism by performing GWASs by using clozapine C/D, norclozapine C/D, and the clozapine/norclozapine ratio as phenotypes, and to further perform meta-analyses that combine our results with data from two previous studies which also investigated clozapine metabolism. Our aim was to validate previous findings by including the Finnish population in an updated meta-analysis, and to perform a smoking-adjusted meta-analysis incorporating the Norwegian cohort.

## Patients and methods

### Patients

Originally, our patient cohort was collected to study adverse effects of clozapine treatment and consisted of 237 patients [21]. Subsample used in this study consists of 170 subjects (101 males/69 females) of North European ethnicity for whom imputed genotype and phenotype data were available (Table 1). Their mean dose of clozapine was 404 mg (SD 149 mg), which is considered to be within the range of standard dose of clozapine [17, 22, 23]. For comparison, the standard deviations for clozapine dose in previous studies by Lind et al. [24] and Reeves et al. [23] have been 167 and 156 mg respectively.

**Table 1:** Description of participants between cohorts.

	Finnish cohort	Norwegian cohort	CLOZUK2
N	170	484	2989
Available covariates	Age, sex, smoking, valproate use	Age, sex, smoking	Age, sex
Multiple laboratory measurements	No, single measurement	Yes	Yes
Sex male/female	101 (59.6%)/69 (40.4%)	298 (61.6%)/186 (38.4%)	2187 (73.2%)/802 (26.8%)
Age	Median 44 years (IQR: 34–52 years)	Median 39 years (IQR: 29–47 years)	Median 43 years (range 18–83 years)
Dose	Mean 404 mg ± 149 mg	Median 400 mg (IQR: 250–500 mg)	Mean 391 mg ± 142 mg
Smoker	51.4%	61.1% (smoking habits known for n = 422/87.2% of patients)	Not available

Our patients were on stabilized clozapine treatment. The majority of them (n = 111) were on clozapine monotherapy without any other antipsychotic medication. The mean serum clozapine concentration in our cohort was 1.50 µmol/l with standard deviation (SD) ± 0.88 µmol/l or 488 ng/l, with SD ± 287.58 ng/l and serum norclozapine level 0.90 µmol/l with SD ± 0.49 µmol/l or 294 ng/l with SD ± 160.13 ng/l. All the patients were diagnosed with a psychotic disorder according to ICD-10 (F2x.xx). Patients with diagnosis of organic psychosis or neurological disorder were excluded. All participants gave written informed consent. The Ethics committee of Satakunta hospital district gave ethical approval for the study and it was done in accordance with Declaration of Helsinki. None of the patients used carbamazapine, a known inducer of several cytochrome P450 enzymes [25, 26]. None of the patients used fluvoxamine, a strong inhibitor of CYP1A2, which might lead to significantly higher concentrations of clozapine [27].

### Laboratory methods

Laboratory samples were collected from 190 patients. Patients were instructed to provide a fasting morning sample, 12 h after taking their evening dose of clozapine, and before taking any morning medication. Each participant provided a 9.0 ml sample of EDTA whole blood, which was stored at −20 degrees Celsius. Clozapine and norclozapine serum concentrations were measured using liquid chromatography at a commercial laboratory.

### Genotyping

Genomic DNA was extracted from peripheral blood leukocytes using a QIAamp DNA Blood Midikit and an automated biorobot M48 extraction (Qiagen, Hilden, Germany). Samples were genotyped using an Illumina Infinium HumanCoreExome-12 DNA Analysis Beadchip, version 1.0., according to the manufacturer’s instructions at Helmholtz Zentrum, München, Germany. The following quality control filters were applied: GenCall score <0.15, GenTrain score less than 0.20, sample and an SNP call rate less than 0.95, Hardy-Weinberg equation P-value less than 10^−6^, excess heterozygosity, cryptic relatedness (pi-hat >0.2), gender check, and multidimensional scaling. After quality control, 176 samples and 531,983 SNPs were available.

Imputation was performed in two stages: haplotype phasing was done using SHAPEIT v2 and genotype imputation using IMPUTE2 v. 2.3.2 and 1000 Genomes Phase I integrated variant set haplotypes as a reference. SNPs with info ≥ 0.3 were considered well imputed. After quality control, 172 samples with imputed genotype were available, and of these samples, data for age, sex and serum concentrations were available for 170 patients, which is the number of patients included in the following analyses.

### GWAS of concentration/dose ratios and clozapine to norclozapine ratio

The clozapine and norclozapine serum levels were divided by the prescribed dose to form the concentration/dose (C/D) ratios. The C/D ratios, along with clozapine to norclozapine ratio, were centered and scaled to have a mean of zero and a standard deviation of one, resulting in Z-scored variables. These three standardized variables were utilized as phenotype outcomes in GWAS. PLINK v2.0 alpha 5 was used for two different linear regression analyses for each outcome. The first model adjusted for age, sex, and the first four genetic principal components (PCs). The second also included smoking and valproate use as covariates.

### Meta-analysis

For the meta-analyses, GWAS results incorporating age, sex, and the first four PCs as covariates were merged with data from CLOZUK2 (patient n = 2989) [15] and Diakonhjemmet (patient n = 484) [17]. The description of participants in the three cohorts and the differences between these cohorts are presented in Table 1. Three meta-analyses were conducted for each outcome on these three combined populations: clozapine C/D ratio, norclozapine C/D ratio and clozapine to norclozapine ratio. Additional three meta-analyses for same outcomes were conducted with our data and that from Diakonhjemmet, adjusting also for smoking and valproate use. CLOZUK2 data was excluded from these additional three meta-analyses on clozapine C/D ratio, norclozapine C/D ratio and clozapine/norclozapine ratio due to the absence of smoking information. In total six meta-analyses were conducted using METAL software (version 2011-03-25) [28], weighting p-values and effect directions by sample size, with genomic control correction applied. Genome-wide significance for SNP associations was defined using a threshold of p < 5 × 10^−8^. The significance threshold for assessing heterogeneity between samples was set at p < 0.05.

## Results

### Meta-analysis of three cohorts without adjusting for smoking

**Fig. 1:**
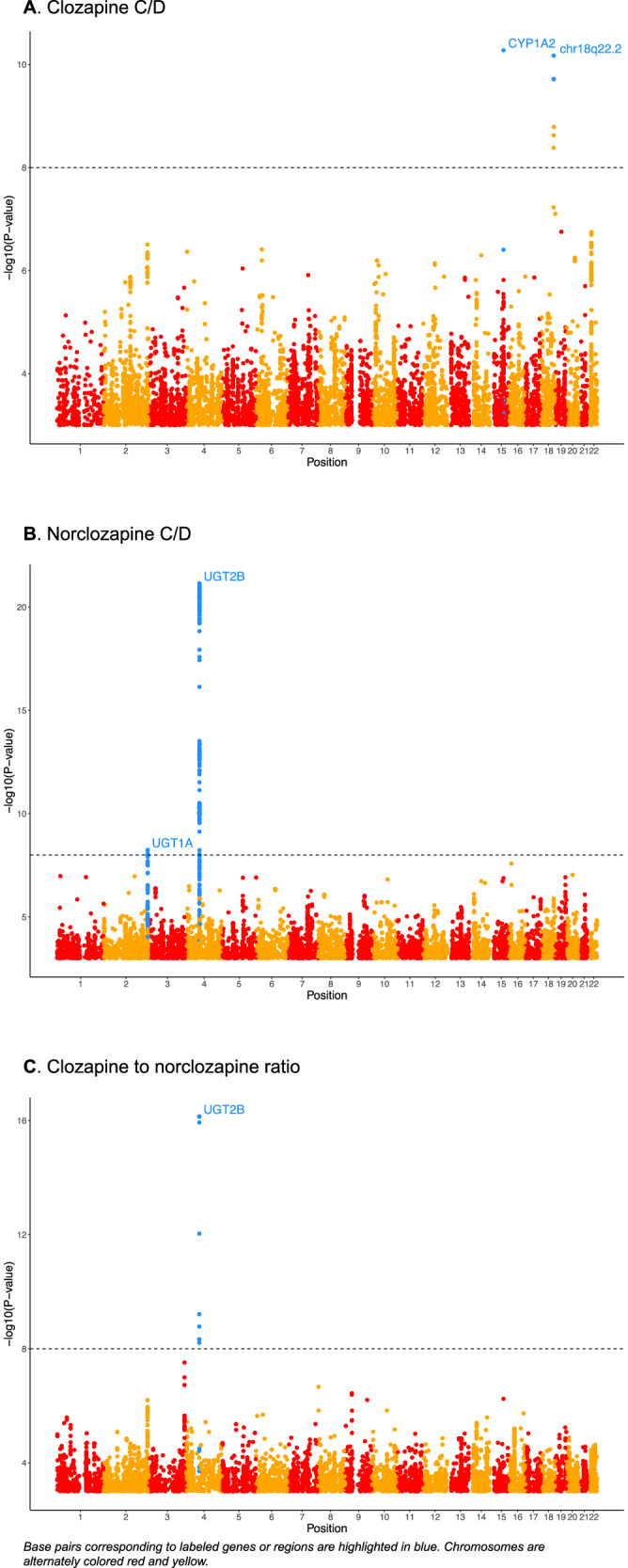
Manhattan plots displaying the genome-wide meta-analysis results across three cohorts. Base pairs corresponding to labeled genes or regions are highlighted in blue. **A** GWAS meta-analysis on clozapine serum concentration dose ratio across three cohorts. **B** GWAS meta-analysis on norclozapine serum concentration dose ratio across three cohorts. **C** GWAS meta-analysis on clozapine norclozapine ratio across three cohorts.

Manhattan plots from meta-analyses of the three cohorts are presented in Fig. 1 and the lead SNPs from loci associated with studied phenotypes are presented in Table 2. The previously found SNP located between *CYP1A1* and *CYP1A2* rs2472297 was significantly associated with clozapine C/D ratio in our meta-analysis (p = 5.27 × 10^−11^, heterogeneity I^2^ = 0, χ^2^ = 0.71, p = 0.40). In our sample, rs2472297 was associated with a 0.31 SD decrease in clozapine C/D. The identified SNPs on chromosome 18 presented in the Fig. 1 were exclusive to our Finnish population and displayed minor allele frequencies (MAF) of 1–2%, indicating that the findings were based on a sample size of only 1–3 patients. Given these frequencies, the results are subject to Type 1 errors but could possibly also point to population-specific mediators in clozapine metabolism.

**Table 2:** Meta-analysis of three cohorts (CLOZUK, Diakonhjemmet Oslo and our sample).

Phenotype	Lead SNPs	Weight (n)	Chr:position	p	Direction**	Heterogeneity	Gene/nearest gene	Previous publications
Clozapine C/D	rs2472297	3152	15:75027880	5.27 × 10^−11^	-?-*	I^2^ = 0, χ^2^ = 0.71, p = 0.40	CYP1A1/CYP1A2 intergenic region	Pardinas et al. [15]
Norclozapine C/D	rs10000284	3631	4:69669183	7.11 × 10^−22^	+++	I^2^ = 65.5, χ^2^ = 5.8, p = 0.055	near UGT2B10 gene region	-
	rs3732218	3636	2:234627304	5.70 × 10^−9^	---	I^2^ = 0, χ^2^ = 0.94, p = 0.63	intron variant UGT1A family gene complex	Samanaite R et al. [33], Yang & Cai et al. [44]
Clozapine to norclozapine ratio	rs6827692	646	4:69930291	7.18 × 10^−17^	++?	I^2^ = 0, χ^2^ = 0, p = 0.98	intron variant UGT2B7	-

*Order from left to right: our sample, Diakonhjemmet Oslo, CLOZUK. -: effect allele is associated with decreased clozapine c/d, + is associated with increased clozapine/cd, ?: data not available.

** In our sample, rs2472297 was associated with a 0.31 SD decrease in clozapine C/D, rs10000284 and rs3732218 with 0.66 and 0.23 SD decreases in norclozapine C/D, respectively, and rs6827692 with a 1.11 SD increase in the clozapine to norclozapine ratio.

**Fig. 2:**
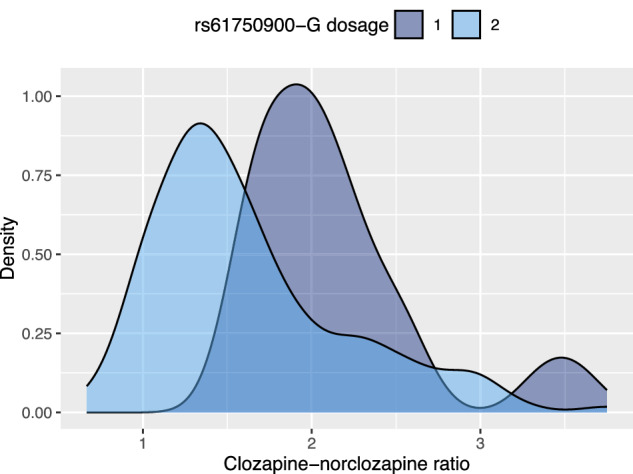
Density plot of UGT2B10 rs61750900 G-allele dosage in relation to clozapine/norclozapine ratio.

The variant that was most strongly associated with norclozapine C/D was rs10000284 (p = 7.11 × 10^−22^, heterogeneity I^2^ = 65.5, χ^2^ = 5.8, p = 0.055). The variant is located near *UGT2B10* gene in chromosome 4 and it has a minor allele frequency of 12% [29]. Among other genome-wide significant variants within this same locus is a missense SNP, *UGT2B10* rs61750900 (4:69681936; PMID: 29438977; p = 1.72 × 10^−21^), which is in perfect linkage disequilibrium with the lead SNP. Thus, we have likely identified the causal variant and the causal gene responsible for the association signal. In the GWAS of the Finnish sample, for rs61750900, the beta was 1.12 for each additional effect allele (p = 3.76 × 10^−12^) indicating a substantial impact on clozapine to norclozapine ratio. A density plot demonstrating the effect of this SNP is presented in Fig. 2.

Also, rs3732218, an intron variant from the *UGT1A* family gene complex, reached genome-wide significance in the three cohort meta-analysis (p = 5.70 × 10^−9^, heterogeneity I^2^ = 0, χ^2^ = 0.94, p = 0.63). The lead SNP in clozapine/norclozapine ratio was rs6827692 (p = 7.18 × 10^−17^, heterogeneity I^2^ = 0, χ^2^ = 0, p = 0.98), an intron variant of *UGT2B7* gene.

### Meta-analysis of two cohorts with adjusting for smoking and valproate use

**Fig. 3:**
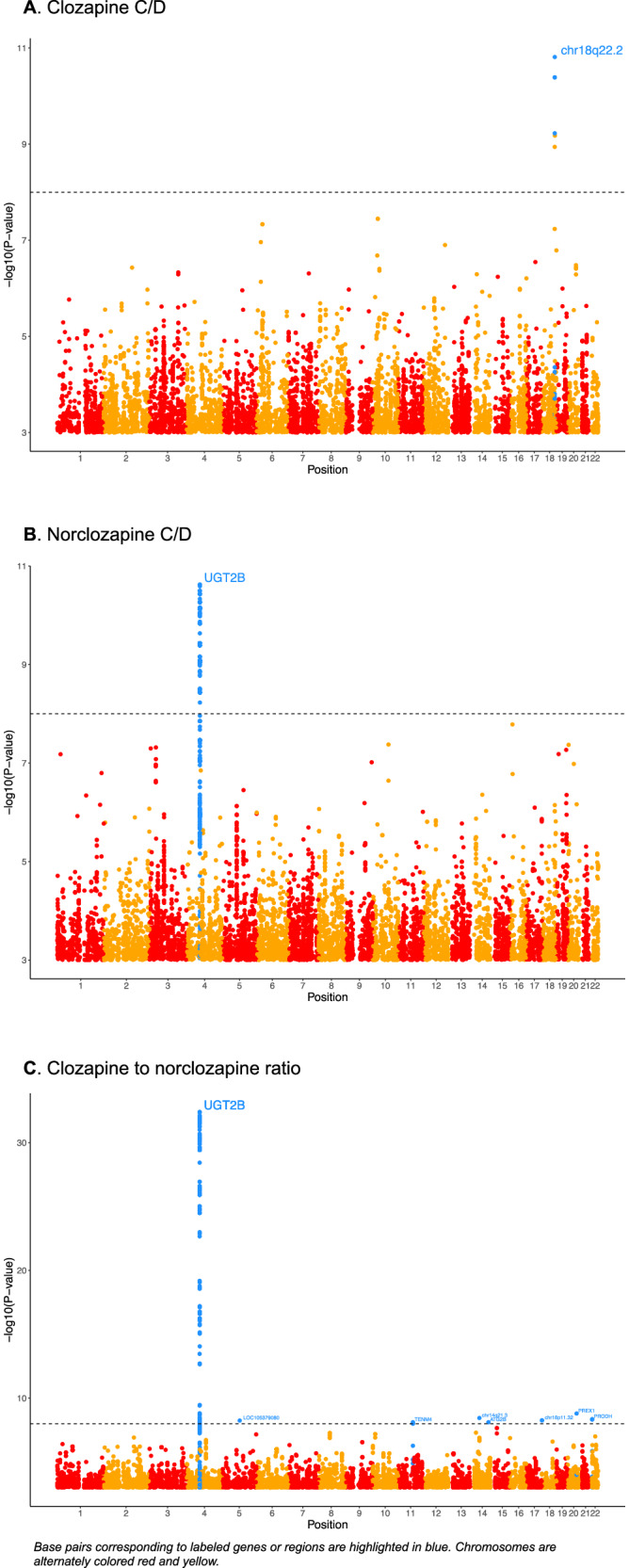
Manhattan plots displaying the genome-wide meta-analysis results across two cohorts. Base pairs corresponding to labeled genes or regions are highlighted in blue. Chromosomes are alternately colored red and yellow. **A** GWAS meta-analysis on clozapine serum concentration dose ratio across two cohorts. **B** GWAS meta-analysis on norclozapine serum concentration dose ratio across two cohorts. **C** GWAS meta-analysis on clozapine norclozapine ratio across two cohorts.

Manhattan plots from meta-analyses of the two cohorts are presented in Fig. 3 and the lead SNPs from loci associated with studied phenotypes are presented in Table 3. For the clozapine C/D ratio, the top SNP was rs1913858266 (p = 1.55 × 10^−11^) a previously unreported SNP found only in our Finnish sample. It exhibited a very low minor allele frequency from chromosome 18 and thus the association should be interpreted with caution.

**Table 3:** Meta-analysis of two cohorts (Diakonhjemmet Oslo and our sample with smoking as covariate).

Phenotype	Lead SNPs	Weight (n)	Chr:position	p	Direction**	Heterogeneity	Gene/nearest gene	Previous publications
Clozapine C/D	rs191385826	163	18:68564011	1.55 × 10^−11^	+?	-	-	-
Norclozapine C/D	rs10000284	576	4:69669183	2.38 × 10^−11^	++	I^2^ = 0, χ^2^ = 0.035, p = 0.85	878277 bytes/nucleotides from UGT2B10 gene region	-
Clozapine to norclozapine ratio	rs835309	580	4:69682855	4.06 × 10^−33^	++	I^2^ = 0, χ^2^ = 0.036, p = 0.55	UGT2B10 intron variant	Wassenaar et al. [35] Guan Y et al. [36]

*Order from left to right: our sample, Diakonhjemmet Oslo, our sample. -: effect allele is associated with decreased clozapine c/d, + is associated with increased clozapine/cd, ?: data not available.

**In our sample, rs191385826 was associated with a 2.43 SD increase in clozapine C/D, rs10000284 with a 0.73 SD decrease in norclozapine C/D, and rs835309 with a 1.11 SD increase in the clozapine to norclozapine ratio.

The top-lead SNP associated with the norclozapine C/D ratio was rs10000284 (p = 2.38 × 10^−11^, heterogeneity I^2^ = 0, χ^2^ = 0.035, p = 0.85) which also emerged as the top SNP in a previous meta-analysis that included the three cohorts. The top SNP in two cohort meta-analysis on clozapine/norclozapine ratio was rs835309 (p = 4.06 × 10^−33^, heterogeneity I^2^ = 0, χ^2^ = 0.036, p = 0.55), a UGT2B10 intron variant.

The previously identified NFIB rs28379954 was not associated with clozapine C/D ratio (p = 0.27 × 10^−6^) in the two cohort meta-analysis. The direction of effect in our sample was opposite. There was significant heterogeneity in rs28379954 between the samples (I^2^ = 89.8%, χ^2^ = 9.78, p = 0.002). In our data, the SNP rs28379954 exhibited a MAF of 4.49%. The genotype distribution, based on hard-called genotypes (n = 167), was as follows: 1.2% were homozygous for the minor allele (CC) and 6.6% were heterozygous (CT).

## Discussion

Our meta-analysis further confirms the association between single nucleotide polymorphism rs2472297 and clozapine C/D, between genetic loci of *CYP1A1* and *CYP1A2*. The association was originally reported by Pardiñas et al. [15] from the dataset we were able to use as part of our meta-analyses. The *CYP1A2* rs2472297 was a significant solitary finding and there were no other significant SNPs in linkage disequilibrium with it.

A missense variant in *UGT2B10* was in strong linkage disequilibrium with norclozapine C/D lead SNP in this locus. A novel SNP rs6827692 from *UGT2B7* intron was associated with clozapine/norclozapine ratio.

The role of *NFIB* SNPs on clozapine C/D could not be replicated in our study. The MAF of *NFIB* rs28379954 was similar in our data as in Diakonhjemmet data (4.5% vs. 4.1%). However, the current meta-analysis is driven by the CLOZUK2 cohort, which represents a ∼6-fold higher sample size than the Diakonhjemmet sample. As the CLOZUK2 cohort lacked information on smoking status, which was essential for detecting an association with NFIB in the Diakonhjemmet cohort [17], a significant NFIB association in the meta-analysis may not be expected. Furthermore, the cohorts included in the meta-analysis have different characteristics, i.e. while the CLOZUK2 and Finnish cohorts were recruited from ordinary clinical practice, the Diakonhjemmet cohort was included from a therapeutic drug monitoring (TDM) database. As a typical indication for performing TDM is lack of effect, the Diakonhjemmet cohort probably contains relatively more patients with low clozapine concentrations. The Diakonhjemmet cohort may therefore be more sensitive in detecting variants encoding increased clearance of clozapine, such as *NFIB* rs28379954 T > C, which has been shown to regulate expression of several drug-metabolizing enzymes (CYP1A2, CYP2D6 and glutathione-S-transferase 1(GST-1)) [18].

*CYP1A1* / *CYP1A2 r*s2472297 has been previously linked with caffeine metabolism [30]. Caffeine is an important substrate for CYP1A2 [31]. This suggests that the effects of rs2472297 on clozapine metabolism are also mediated through CYP1A2. The variant was missing from the Norwegian sample and therefore this association could not be replicated in previous study by Løvsletten Smith et al. [17]. Our two-cohort meta-analysis did not identify any novel SNPs affecting the clozapine C/D ratio.

Norclozapine C/D has previously been linked to *UGT2B10* loci by Pardiñas et al. [15] and by Løvsletten Smith et al. [17]. Our lead SNP in this locus (rs10000284 in linkage disequilibrium with rs61750900) supports the central role of the *UGT2B* family in norclozapine metabolism. This missense variant has been linked to absolute neutrophil count (ANC) on clozapine patients in CLOZUK2 population, with long term exposure of high levels of clozapine, but also high clozapine to norclozapine ratio lowering absolute neutrophil count [32]. Also, rs3732218, an intron variant from *UGT1A* family gene complex reached significance in the three cohort meta-analysis for norclozapine C/D. This SNP was previously reported by Samanaite et al. as one of the SNPs related to biological predictors of clozapine response [33].

Pardiñas et al. found a SNP affecting clozapine to norclozapine metabolic ratio that was possibly linked to *UGT2B7* area [16]. Clozapine to norclozapine metabolic ratio was affected by SNP rs6827692, an *UGT2B7* intron area variation in our three cohort meta-analysis but this SNP was not available in CLOZUK2 population. Valproate use, and smoking were not used as covariates in our three cohort meta-analysis due to the lack of this data in the CLOZUK2 dataset. Interestingly, the role of certain *UGT2B7* polymorphisms in valproate metabolism has been discussed recently, suggesting this is another shared metabolic route for clozapine metabolites and valproate [34].

The top SNP in two cohort meta-analysis on clozapine to norclozapine ratio was rs835309, *UGT2B10* intron variant, that was previously reported in conjunction with *UGT2B* genotypes affecting nicotine and nitrosamine glucuronidation in European and African American smokers [35] and in conjunction with SNPs affecting dexmedetodimine spectrometry samples in pediatric patients [36].

The role of *UGT*-families in norclozapine metabolism opens interesting possibilities on studying interactions [37, 38] and adverse effects associated with co-medication of clozapine and valproate [39, 40]. Additionally, the impact of various *UGT* polymorphisms on the formation of specific clozapine N-metabolites, some of which are associated with agranulocytosis [12, 41], presents a significant area of interest. Further research on this area could help in understanding the mechanisms behind agranulocytosis, the adverse effect of clozapine that typically raises the most concern, especially in light of recent discoveries about immunological phenotypes related to this condition [42, 43]^.^

We detected an association between clozapine C/D ratio in chr18q22.2 locus. This association could point to population-specific mediators in clozapine metabolism. Considering the small sample size, this result should however be interpreted with caution as we couldn’t identify any biological mechanisms mediating the effects of the locus.

The strengths of our study were well documented clozapine doses, co-medications such as valproate and smoking status among our patient cohort. The use of two other major patient cohorts (Diakonhjemmet, CLOZUK2) in meta-analyses made this one of the largest patient populations in recent studies on clozapine metabolism.

Weaknesses in our study are the cross sectional, single laboratory measurements and relatively small numbers in our own cohort. Additionally, compliance with the instructions for fasting and withholding morning medications was not confirmed. Furthermore, the absence of rs2472299 data in the *CYP1A1/1A2* intragenic region from the Diakonhjemmet population limits our understanding of the role of this SNP in clozapine metabolism among smokers and valproate users. All the study populations were of North European ethnicity, which may limit the generalizability of the findings to other ethnic groups. No data on treatment response was available in any of the cohorts, nor of possible adverse effects of clozapine treatment were available in Diakonhjemmet- and CLOZUK2-cohorts. Selection bias may have occurred due to patients discontinuing medication as a result of severe or intolerable adverse effects.

In conclusion, our meta-analysis further validates the link between rs2472297, situated between *CYP1A1* and *CYP1A2*, and the clozapine C/D ratio. No association was observed between *NFIB* gene polymorphisms and the clozapine C/D ratio in this study. We found that a locus near the *UGT2B10* gene correlates with the norclozapine C/D ratio, with rs61750900 likely being the causal variant. Furthermore, we identified a novel SNP, rs6827692, within the *UGT2B7* intron, which influences the clozapine to norclozapine ratio. The role of glucuronidation pathways and different clozapine metabolites deserve further studies to develop better clinical tools for predicting benefits and risks of using this complex drug.

## Data Availability

GWAS summary statistics of the Finnish cohort are available from the corresponding author upon reasonable request.
